# Exploring 70 Years of the British National Health Service through Anniversary Documents

**DOI:** 10.15171/ijhpm.2018.21

**Published:** 2018-03-06

**Authors:** Martin Powell

**Affiliations:** Health Services Management Centre, School of Social Policy, University of Birmingham, Birmingham, UK.

**Keywords:** National Health Service, UK, 70th Anniversary, Documents, Content Analysis

## Abstract

The British National Health Service (NHS) celebrates its 70th birthday on July 5, 2018. This article examines this anniversary through the lens of previous anniversaries. It examines seven documents close to each anniversary over a period of some 60 years, drawing on interpretive content analysis, based on the narrative dimensions of context (structure and finance); success or achievements; problems; and solutions or recommendations. It finds that the anniversary documents tend to show change rather than consistency. For example, the Guillebaud Report tended to dismiss the problem of ageing populations, for it to reappear in 1979 and 1989, to fade in 2009, and reappear once more in 2017. Despite being downplayed or ignored in some years, the problems identified by most of the documents such as demography and technology are unlikely to disappear. Some solutions such as market-based reform have flowed and ebbed over the years, and the ‘solution’ of structural reorganisation in one year has become the ‘problem’ in a future year. While predicting the future is always hazardous, it can be said with some confidence that future anniversaries are likely to see discussion of similar themes.

## Introduction

The British National Health Service (NHS) celebrates its 70th birthday on 5 July 2018 (https://www.england.nhs.uk/nhs70/). This article examines this anniversary through the lens of previous anniversaries**.** Pollitt^1^ adopted a narrative approach to analyse the scope, dominant themes, proffered solutions, evidence base, key assumptions and style and presentation of ﬁve key UK public management reform white papers over a period of 41 years. His unit of analysis was the White Paper – an ofﬁcial document stating government policy. Each White Paper was treated as a narrative, with his core questions concerned with what the story was and how it was told. He stressed that white papers are a particular kind of story: tales of unsatisfactory pasts and better futures, or ‘advocacy narratives’ with ‘happy endings.’

His analysis took the ‘long view,’ looking for both changes and continuities over time. He argued that the longitudional frame or long-term perspective had too often been sidelined in the public management literature, usually in favour of a tight focus on the latest developments, and on cross-sectional comparisons.

This article differs from Pollitt^1^ in two main ways. First, it selects documents from as close to decennial anniversaries as possible. Second, this means that the documents are more varied than white papers, including green (consultative) and white papers, but also advisory committees and Royal Commissions. The documents are: Guillebaud^2^; Ministry of Health^3^; Royal Commission on the NHS^4^; Working for Patients^5^; The NHS. Modern. Dependable^6^; From Good to Great^7^; and The Long-term Sustainability of the NHS and Adult Social Care.^8^ Details of the broader context can be found in Klein^9^ and Timmins.^10^

This article draws on interpretive content analyses that includes attention to both manifest and latent content, and centres on descriptive narratives, or themes, summarizing the collected and coded data.^11^ The coding was a mixture a priori or deductively generated coding (cf Pollitt^1^) and inductive or “emergent” coding. Put another way, it started with Pollitt’s themes of scope, dominant themes, proffered solutions, evidence base, key assumptions and style and presentation. However, it adapted this by developing a coding list from the first few documents by selecting a tentative list of topics that were found to be revealing or useful. In addition to key words (cf Pollitt^1^), it drew on connotative codes, which are based not on explicit words but on the overall or symbolic meaning of phrases or passages. This gives the narrative dimensions of context (structure and finance); success or achievements; problems; and solutions or recommendations. Due to the varied nature of the documents, not all dimensions are present. For example, the 1968 green paper is concerned with structure. Moreover, some of the dimensions can blend into one another. For example, finance can be seen as a problem.

### Tenth Anniversary

A Debate in the House of Commons to mark the tenth anniversary of the creation of the NHS was an ‘exercise in mutual self-congratulation as Labour and Conservative speakers competed with each other in taking credit for the achievements of the NHS.’^9^ Similarly, according to Timmins^12^ it was broadly positive, and a ‘matter of quiet congratulation.’ However he continued that it was calm only because the NHS had just weathered the first of its many major crises. The Guillebaud Committee was set up in 1953 by the Conservative government after NHS expenditure had far exceeded estimates. This committee was similar in some ways to a Royal Commission in that it asked independent experts to examine the NHS. Although the term ‘cost’ appeared in the title, the committee interpreted its terms of reference fairly widely, and examined other issues such as structure.

#### Guillebaud (1956)

The Committee noted that the current net cost of the NHS as a percentage of gross national product (GNP) initially rose from 3.51% in 1948-1949 to a peak of 3.75% in 1949-1950 before falling to 3.24% in 1953-1954 (p. 9). In a comparison with pre-war capital expenditure, capital expenditure in 1938-1939 was more than three times as high as it was in 1952-1953 (p. 33). Overall, the proportion of national investment devoted to the NHS has been very small indeed throughout the period and has never reached even 1% (p. 31).

The Committee then turned to estimating the future funding of the NHS. It cited the Registrar General’s 1953 estimates: ‘there is no justification for the alarm that has been expressed about the impact of an ‘ageing population’ on the cost of the NHS. Changes in age structure by themselves are calculated to increase the present cost of the Service to public funds by 3 ½ per cent by 1971/1972. The Registrar General stated that one reason why the additional costs of more old people are insignificant is because only about one-fifth of expenditure is currently devoted to this age group. A second explanation is that the old group is currently receiving a lower standard of service than the main body of consumers (p. 40). The Committee appeared to accept this analysis: population changes *in themselves* are not likely to exert a very appreciable effect on the future cost of the NHS (p. 49).

The Committee then turned to the general structure of the NHS. First, it considered the definition of an ‘adequate service’ (p. 49). It stated that it is ‘clearly inadequate now in the sense of meeting every demand justifiable on medical grounds.’ The advance of medical knowledge and rising public expectations continually placed new demands of the Service. ‘We conclude that in the absence of an objective and attainable standard of adequacy the service must … provide the best service possible within the limits of the available resources’ (p. 50).

The Committee was very conscious of the fact that the NHS had only been operating for seven years. Despite certain weaknesses, the Service’s record since 1948 had been one of ‘real and constructive achievement.’ It considered proposals for radical reorganisation from the 1948 ‘Tri-Partite’ system that essentially was based on the three existing branches of health service administration (see below): Executive Councils (bodies concerned with local primary care professionals such as general practitioners [GPs]); Hospitals (under some 14 regional hospital boards [RHBs] and 330 local hospital management committees [HMCs])); and elected multi-purpose local authorities that were concerned with public health and community nursing. The four proposals were: one local authority for all branches; hospitals transferred to the local authorities; transferring the work of the Executive Councils to the local authorities or RHBs; and a central National Board or Corporation. However, ‘we believe that unless an overwhelming case could be made out for any basic reorganisation of the Service, it would be in the best interests of the Service to leave the present administrative structure undisturbed (p. 53). The Report did not number its ‘Recommendations.’ However, most of these seem to be relatively minor in nature, while there are a large number of endorsements of the status quo: ‘we do not recommend….’

### Twentieth Anniversary

According to Klein,^9^ ‘if the first decade of the NHS was the period of consolidation, the next decade and a half was a period of innovation.’ This period saw the Hospital Plan of 1962, with the 15 years from 1960 to the mid-1970s a period of rapid growth in public expenditure (p. 49). However, the period also saw the first major reorganisation of the NHS. ‘In search of the organisational fix,’ Minister of Health Kenneth Robinson published the first consultative document on reorganisation in 1968, which was published to coincide with the NHS 20th anniversary.^13^ As the title suggests, the main focus was on the administrative structure of the NHS, and it was a ‘green’ or consultative paper.

#### Ministry of Health (1968)

It was stated that while the 1948 framework was well suited to the immediate needs of the NHS, there was now widespread recognition that the time had come for that structure as a whole to be radically reconsidered. The proposals were ‘entirely tentative,’ and subject to consultation. The NHS required the closest collaboration between doctors, nurses and other workers. ‘The central theme of this Green Paper must be the unified administration of the medical and related services in an area by one authority, in place of the multiplicity of authorities concerned in the present arrangements’ (p. 6). It recognised that hospital authorities were numerous: 14 RHBs, 330 HMCs and 36 Boards of Governors for Teaching Hospitals, and health care in the local community was a divided responsibility: 134 Executive Councils and 175 local health authorities (HAs), and welfare services provided by local authorities. This ‘Tripartite’ structure was complex, consisting of two systems of finance, and varying population size. For example, there were 25 local HAs under 75 000 population but 6 with over 1 million. In short, the number of separate authorities in the present administrative structure was nearly 700, with wide variations in size, resources, opportunity and scope. It suggested about 40-50 Area Boards, with advantages of having a broadly similar pattern to local government.

### Thirtieth Anniversary

Klein^9^ argued that if the start of the 1970s saw the apotheosis of paternalistic rationalism, with the 1974 reorganisation as its monument, the second half of the decade produced the politics of disillusionment. As a result of the 1973 economic crisis, the average increase in the NHS budget of 4.3% under the 1970-1974 Conservative Government, but 1.5% under the 1974-1979 Labour Government. Moreover, the second half of the 1970s was a period of medical militancy and of trade union militancy. For the first time in the history of the NHS, doctors took industrial action, resulting in ‘the politics of ideological confrontation’ over pay beds (p. 85). Klein also pointed to ‘the politics of organisational statis’ and sense of crisis that led to the setting up of a Royal Commission on the NHS (p. 90; cf Timmins^12^). Klein noted that the Report both reflected the growing disillusionment and represented an attempt to maintain the consensus. It reaffirmed the basic philosophy of the NHS, and delivered an overwhelming – though not uncritical – endorsement of the NHS. In this respect it resembled the Guillebaud Report, but while that virtually silenced political argument about the NHS for 10 years, the Royal Commission marked on the contrary the beginning of a new debate, with an incoming Conservative government of 1979.

#### Report of the Royal Commission on the NHS (1979)

It opened by stating that it was appointed at a time when there was widespread concern about the NHS: a reorganisation which few had greeted as an unqualified success; industrial disputes; and a chill economic climate (p. 1). It noted that only Guillebaud had considered the NHS as a whole (p. 2). It stated that there was general agreement in evidence that the structure of the NHS needed slimming (p. 321), and it was considered that there is one management tier too many in most places (p. 325). The 1982 NHS reorganisation saw the area HAs created only in 1974 abolished, with the single tier below regional level being the District Health Authority (see eg, Timmins^10^). Like the Guillebaud Report, it argued that there was no objective or universally acceptable method of establishing what the ‘right’ level of expenditure on the NHS should be, but on balance their recommendations would increase the cost of the service (p. 353). It stated that its evidence contained a complete spectrum of descriptions of the present state of the NHS ranging from ‘the envy of the world’ to its being ‘on the point of collapse.’ Its judgement lay between these extremes (p. 13). In terms of international comparisons, it noted that the United Kingdom spent less than most over developed countries, and also performed relatively poorly in terms of indicators of health such as life expectancy, and perinatal and maternal mortality. It concluded ‘we need not seem ashamed of our health service and there are many aspects of it of which we can be justly proud’ (p. 27). It noted that ‘a common criticism is that the NHS is a sickness service rather than a health service’ (p. 35). It stated that easily the most popular remedy for the failings of the NHS, especially and understandably with those working in it, was that much more money should be made available. Others included alternative methods of financing (eg, charges); the NHS should be taken out of politics; integrating health and personal social services; and further NHS reorganisation (pp. 35-6). In contrast to Guillebaud, it suggested that demographic change of the growing number of old people and particularly those over 75, will be the greatest single influence on the shape of the NHS for the rest of the century (p. 379). In total it made 117 recommendations, but few of great or lasting significance.

### Fortieth Anniversary

According to Timmins,^12^ the worst financial crisis in the NHS’s history led to the Presidents of the three senior Royal Colleges appeal in public to the Conservative Government to “save our NHS.” This contributed towards the 1988 NHS Review, which led to the White Paper, ‘Working for Patients,’^5^ and to the 1991 internal market. Klein^9^ characterised the period as ‘the politics of value for money’ (Ch 5) which led to ‘the politics of the big bang’ (Ch 6) of the 1989 White Paper that marked the ‘end of consensus on the NHS’ in the biggest explosion of political anger and professional fury in the history of the NHS (p. 105). Put another way, the Presidents should perhaps have been careful what they wished for. They clearly saw ‘saving’ the NHS in terms of more money, but the Conservative Thatcher government introduced the ‘internal market’ or ‘purchaser/provider split’ which has been termed the biggest change in the history of the NHS, and shaped the policy direction of the NHS for the next twenty five years or so.

#### Working for Patients (1989)

It was argued that the ‘structure’ of the NHS required change, but at face value, the document appears to be fairly anodyne. For example, it does not use term ‘internal market’ or ‘purchaser/provider split,’ although those terms were used by both sides in a Parliamentary Debate just before the document’s publication (Hansard House of Commons 1988). Moreover, it reaffirmed the principles of the NHS: the NHS is, and will continue to be, open to all, regardless of income, and financed mainly out of general taxation [albeit a minimalist interpretation]. Turning to finance, it was stated that total gross expenditure on the NHS increased from some £8 billion in 1978-79 to £26 billion in 1989-1990, an increase of 40% after allowing for general inflation (p. 2). It stated that the NHS was growing at a truly remarkable pace: staff, expenditure, activity such as in-patients, and throughout the 1980s the Government presided over a massive expansion of the NHS. The achievements of the NHS included high standards of health care, and longer lengths and quality of life. But – ‘the need for change’ – it had become increasingly clear that more needed to be done because of rising demand and an ever-widening range of treatments made possible by advances in medical technology (pp. 2-3). It was clear that the organisation of the NHS needed to be reformed. The Government wished to raise the performance of all hospitals and GP practices to that of the best. It was convinced that it can be done only be delegating responsibility as closely as possible to where health care is delivered to the patient (p. 3). It set out seven key measures: delegation of power and responsibility; self-governing hospitals of NHS Hospital Trusts; money able to cross administrative boundaries; 100 new consultant posts; large GP practices able to hold budgets; management bodies reduced in size and reformed on business lines; and medical audit (pp. 4-5). The central aims were to: to extend patient choice; to delegate responsibility to those who are best placed to respond to patients’ needs and wishes; and to secure the best value for money. The Government would build further on the strengths of the NHS, while tackling its weaknesses. This would ensure that the NHS becomes an even stronger, more modern Service, more committed than ever to working for patients (p. 101).

Although the term ‘market’ was not used, it discussed several key ingredients of a market. For example, an NHS Hospital Trust will earn its revenue from the services it provides, with the main source of revenue from contracts (p. 24). Incentives were stressed: the practices and hospitals which attract the most custom will receive the most money (p. 48). The public and private sectors should working together. The Government expected to see further increases in the number of people wishing to make private provision for health care, and so allowed tax relief for retired people (pp. 6-7). There was already a growing partnership between the NHS and the independent health sector. The Government believed there was considerable scope for building on these initiatives: GPs and HAs would be able to use NHS funds to pay for some private sector treatments ‘if this offered better quality or better value for money than buying NHS services’ (p. 68). The Government believed that there was scope for much wider use of competitive tendering, beyond the non-clinical support services which formed the bulk of tendering so far. This could extend as far as the wholesale ‘buying in’ of treatments for patients from private hospitals and clinics, which had proved effective under the Government’s waiting list initiative (p. 70). Taken together the reforms amounted to ‘the most significant review of the NHS in its 40-year history’ (p. 100).

### Fiftieth Anniversary

During the 1997 Election Labour claimed that the nation had ‘14 days to save the NHS.’ The Labour landslide saw ‘the politics of the third way,’^9^ with the production of a White Paper some six months later.

#### The NHS. Modern. Dependable (1997)

The White Paper contained a Foreword by Labour Prime Minister Tony Blair that stated that **‘**creating the NHS was the greatest act of modernisation ever achieved by a Labour Government.’ However, the NHS needed to modernise in order to meet the demands of today’s public. In short, he wanted the NHS to become a modern and dependable service that is once more the envy of the world. Problems included long waiting lists; variable quality; and an NHS that treated people when they were ill rather than one that worked with others to improve health and reduce health inequalities. The document rejected those who argued that the NHS could not accommodate these pressures and would need huge increases in taxation, a move to a charge-based service, or radical restrictions in patient care. ‘So do the public.’ It argued that the pressures on the NHS were- and always have been- exaggerated. It noted that Bevan stated some 50 years ago that “expectations will always exceed capacity.” It continued that ‘Likewise demographic pressures can be overstated.’ However, the NHS had to change. It had to modernise to meet the demands of a new century. The document stated that the internal market would be replaced by a system of ‘integrated care,’ based on partnership and driven by performance, where cooperation would replace competition. It formed the basis for a ten year programme to renew and improve the NHS through evolutionary change rather than organisational upheaval. These changes would build on what has worked, but discard what has failed. However, although it would not mean a wholesale structural upheaval, new institutions such as new local commissioners of Primary Care Groups would be created. At the national level, there would be new institutions such as the National Institute for Clinical Excellence (to give a strong lead on clinical and cost-effectiveness), the Commission for Health Improvement (to support and oversee the quality of clinical services at local level, and to tackle shortcomings) and National Service Frameworks. The Government stated it would raise spending in real terms every year. The Government committed itself anew to the historic principle of the NHS: that if you are ill or injured there will be a national health service there to help; and access to it will be based on need and need alone. The White Paper aimed to renew the NHS as a one-nation health service offering fairness and consistency to the population as a whole, with improvements in quality and efﬁciency; speed of access to care; and improved health status and reduced health inequalities. But 3 years into a 10-year plan, after a change of Secretary of State, the NHS Plan of 2000 reversed direction, ramping up the market that Labour had claimed to abolish only some 1000 days earlier.

### Sixtieth Anniversary

‘The politics of transition’^9^ covered the Brown premiership, which was a period of transition from political stability to political uncertainty, from an era of optimism about the economic future to one of anxiety, and also a period of transition for the NHS: from market creation to market shaping. It produced a ‘pandemic of visions’ (p. 253) such as the Darzi Report,^14^ but also moves ‘towards the fiscal ice age’ (p. 256). Timmins^12^ regards the NHS at 60 as the ‘calm before the storm.’ He noted that the Darzi Report set out the set of challenges facing the NHS in the 21st century: rising expectations; demand driven by demographics; the continuing development of our ‘information society’; advances in treatments; the changing nature of disease; and changing expectations of the health workplace. A White Paper of 2009 built on and implemented Darzi’s vision.

#### NHS 2010–2015: From Good to Great (2009)

The document began in celebratory mode: 15 years ago, the NHS had sunk to such a low ebb that many voiced doubts over its long-term survival. It was a huge turnaround in fortunes and a great success story. It had gone from struggling to generally good, but a new ambition would take it ‘from good to great’ implementing our vision of a preventative, people-centred, productive NHS. The document pointed to a decade of record, sustained investment which meant that funding doubled in real terms over the last 12 years and was almost exactly the average among the Organisation for Economic Co-operation and Development (OECD) countries. It stated that the following year, the NHS would receive a substantial increase in funding and the Pre-Budget Report has confirmed that this uplift will be locked in to frontline budgets for the 2 years that follow. All this meant that the NHS had made huge progress over the last decade. Care had improved, and NHS waiting times were the shortest they have been since NHS records began. Moreover, the NHS, and the values it proclaims to the world, was one of the best things about Britain today. First and most important, it is founded on strong values that bind us all together. The NHS will continue to be based on these values and provide care based on need and not on ability to pay.

Almost as an after-thought, the elephant in the room was mentioned: finding some £15­20 billion in ‘efficiency savings’ over the 3-year period from April 2011. The tariff payment system would have a maximum uplift of 0% for the next 4 years, which will ‘drive all providers to become as efficient as the highest performers’ (p. 51). There was no mention of the other elephant in the room of the ‘Mid Staffordshire’ scandal (see Klein^9^). However, a change in Government in the 2010 Election led to a very different vision and the ‘the politics of confrontation’ over the 2010 White Paper ‘Equity and Excellence.’^9^

### Seventieth Anniversary

The 70th anniversary is set within the longest financial squeeze in the history of the NHS, and widespread concerns of ‘crisis.’

#### The Long-term Sustainability of the NHS and Adult Social Care (2017)

It stated that our NHS was in crisis and the adult social care system was on the brink of collapse. ‘Our conclusion could not be clearer. Is the NHS and adult social care system sustainable? Yes, it is. Is it sustainable as it is today? No, it is not. Things need to change.’

It noted that the NHS had survived a long series of crises since its foundation. Accusations of underfunding, back-door privatisation and unnecessary reorganisations, have plagued successive Secretaries of State for Health. Many witnesses portrayed an NHS at breaking point. However, ‘this crisis is different from the other crises.’ It concluded that ‘whatever short-term measures may be implemented to muddle through today, a better tomorrow is going to require a more radical change.’

While the NHS had evolved considerably since 1948, the drivers of change—from demographic factors and changing disease patterns, to technological and medical advances, income effects and increasing relative health care costs—were intensifying at a relentless pace and fuelling rising public expectations. The system, which was originally designed to treat short-term episodes of ill health was now caring for a patient population with more long-term conditions, more co-morbidities and increasingly complex needs. It noted that in comparative terms, the United Kingdom had historically spent less on health when compared with other nations, has fewer hospital beds, fewer doctors and fewer nurses per head, and often worse outcomes for survival from stroke, heart attacks and many cancers. Other problems included a culture of short-termism; low productivity; wide variations in provider performance; slow adoption of new technologies; lack of integration between health and social care; and a lack of focus on prevention. The Commission made 34 Recommendations including that health spending beyond 2020 should increase at least in line with the growth of gross domestic product (GDP); service transformation; and the establishment of an Office for Health and Care Sustainability.

## Discussion and Conclusions

Writing for the 60th Anniversary of the NHS, Timmins^12^ stated that his brief history of the big anniversaries demonstrates ‘plus ça change’ – that many of the issues that the NHS is grappling with right now, and will continue to grapple with, always have been there. For example, he claimed that the pressures first clearly spelled out more than 50 years ago in the Guillebaud report – ageing populations, the cost of technological advance and rising expectations – remain.

The Anniversary documents (Table) suggest some continuities. For example, paralleling some of Pollitt’s conclusions, these include the elusiveness of the impacts of management change, with none of the documents offering a clear set of targets or yardsticks by which ‘success’ or ‘failure’ could subsequently be judged, and the thinness of evidence underpinning the reforms proposed. Other ‘constants’ include affirmations of the principles of the NHS. For example, as the House of Lords Commission^8^ put it, we strongly recommend that a tax-funded, free-at-the-point-of-use NHS should remain in place as the most appropriate model for delivery of sustainable health services both now and in the future. However, the NHS has seen some version of ‘Groundhog Day’ in that for many years reports have stressed the visions such as integrated, seamless and more person-centred care; more care delivered in primary and community settings, and a greater focus on prevention. Moreover, many of these visions are common to other health care systems. For example, according to the Parliamentary Review of Health and Social Care in Wales,^15^ the vision for care that Wales should achieve is one being pursued by most developed nations in the face of similar circumstances. This is to revolutionise care so that it empowers individuals to take decisions, tailors care to the individual’s expressed needs and preferences, is far more proactive and preventative, is provided as close as possible to people’s homes, is seamless, and is of the highest quality. Its High Level Recommendations include: Bold New Models of Seamless Care; Put the People in Control; Harness Innovation, and Accelerate Technology and Infrastructure Developments; A Health & Care System that’s always learning. However, it stated that ‘nobody we spoke to during the course of this Review disagreed with our assessment that the case for change is compelling.’ It then posed the question: ‘If the case for change is compelling, then why hasn’t it compelled?’ (p. 5).

**Table:** The Anniversary Documents

**Anniversary**	**Period (Klein 2013)**	**Government**	**Key Document**	**Key Themes**
1958	Consolidation	Conservative (1951-1964)	Guillebaud (1956)	**Structure**: No case for major reorganisation **Finance**: NHS not out of financial control; required more investment **Achievements**: Record of real and constructive achievement **Problems:** Little concern over population ageing **Solutions:** Many Recommendations, but few major ones
1968	Technocratic Change	Labour (1964-1970)	National Health Service: the Administrative Structure of the Medical and Related Services in England and Wales (1968)	**Structure**: Tri-partitite system **Problem**: Lack of integration; structure needs to be radically reconsidered **Solutions**: Central theme of unified administration in an area by one authority
1978	Disillusionment	Labour (1974-1979)	Report of the Royal Commission on the National Health Service (1979)	**Structure**: Needed slimming, with one management tier too many in most places **Finance**: Difficult to establish the ‘right’ level of expenditure, but on balance recommendations would increase the cost of the service **Achievements**: Largely positive, but poor international comparisons **Problems**: Ageing **Solutions**: 117 Recommendations, but few of great or lasting significance
1988	Value for money/the Big Bang	Conservative (1979-1997)	Working for Patients (1989)	**Structure**: New institutions of Hospital Trusts and GPFH introduced within an ‘internal market’ **Finance**: Real increase in expenditure of about 40% over 10 years **Achievements**: High standards of health care, and longer length and quality of life. **Problems**: Lack of incentives **Solutions**: Internal market
1998	Third Way	Labour (1997-2010)	The New NHS (1997)	**Structure**: No wholesale structural upheaval, but some new national and local institutions **Finance**: The Government stated it would raise spending in real terms every year **Achievements**: The historic principle of the NHS with access based on need and need alone **Problems**: Demographic pressures can be overstated **Solutions**: Internal market replaced by a system of 'integrated care'
2008	Transition	Labour (1997-2010)	From Good to Great (2009)	**Structure**: No change suggested **Finance**: NHS expenditure had doubled in real terms since Labour took office in 1997 **Achievements**: Celebratory and optimistic; NHS based on strong values; recent positive record **Problems**: Downplayed Darzi challenge of demographic change; coming austerity; and scandal **Solutions**: Vision of a preventative, people-centred, productive NHS
2018	NA	Conservative/ Liberal Democrat Coalition (2010-2015); Conservative (2015-2016); Minority Conservative (2016-)	The Long-term Sustainability of the NHS and Adult Social Care (2017)	**Structure**: Lack of integration between health and social care **Finance**: Insufficient funding; ‘crisis’ point **Problems**: Demographic challenge; relatively poor international comparisons **Solutions**: 34 Recommendations

Abbreviations: GPFH, general practitioner fund holding; NHS, National Health Service; NA, not applicable.

However, in the main, the anniversary documents tend to show change rather than consistency. For example, contrary to Timmins^12^ claims, the Guillebaud Report tended to dismiss the problem of ageing populations, for it to reappear in 1979 and 1989, to fade in 2009, and reappear once more in 2017. While some tend to stress positive internal evaluations (sometimes more related to principles than delivery) (eg, Guillebaud,^2^ Royal Commission^4^), others pointed to more negative external comparisons (eg, Royal Commission^4^; House of Lord^8^). The Royal Commission of 1979 seemed to provide a two way bet of a largely positive evaluation; but relatively poor comparative performance. While a more modern service in 1989 meant a ‘market,’ modernisation in 1997 meant abolishing the market in favour of integrated care. However, Guillebaud^2^ discussed and rejected solutions to increase integration, and the 1968 Green Paper had a ‘central theme of unified administration.’ While Guillebaud considered that it was too early to consider reorganisation, a document 12 years later searched for the ‘organisational fix,’ that has led to an almost continual search for the perfect structure since that time that has featured some of the solutions discussed but rejected by Guillebaud.^2^

Despite being downplayed or ignored in some years, the problems identified by most of the documents such as demography and technology are unlikely to disappear. Some solutions such as market-based reform have flowed and ebbed over the years, and the ‘solution’ of structural reorganisation in one year has become the ‘problem’ in a future year. While predicting the future is always hazardous, it can be said with some confidence that future anniversaries are likely to see discussions of similar themes.

In conclusion, and in terms of the narrative dimensions, there has been little agreement on structure over the 70 years, but a broad agreement that more expenditure is required (but a lack of clarity of how much). While there is some continuity in discussions of achievements, problems such as demographic challenges appear to vary over time. Perhaps most importantly, solutions exhibit both continuity and change. However, while some solutions such as marketisation have ebbed and flowed over time, other solutions such as prevention and care closer to home are hardy perennials, but this merely suggests that we are little closer to implementing these long advocated solutions.

## Ethical issues

Not applicable.

## Competing interests

Author declares that he has no competing interests.

## Author’s contribution

MP is the single author of the paper.
